# Identification of Differentially Expressed Non-coding RNA Networks With Potential Immunoregulatory Roles During *Salmonella* Enteritidis Infection in Ducks

**DOI:** 10.3389/fvets.2021.692501

**Published:** 2021-06-16

**Authors:** Yu Zhang, Xiaoqian Dong, Lie Hou, Zhengfeng Cao, Guoqiang Zhu, Wanwipa Vongsangnak, Qi Xu, Guohong Chen

**Affiliations:** ^1^Joint International Research Laboratory of Agriculture and Agri-Product Safety of Ministry of Education, Yangzhou University, Yangzhou, China; ^2^Jiangsu Key Laboratory of Zoonosis, Yangzhou University, Yangzhou, China; ^3^Department of Zoology, Faculty of Science, Kasetsart University, Bangkok, Thailand

**Keywords:** duck, ceRNA, non-coding RNA, *Salmonella* Enteritidis, immune regulation

## Abstract

*Salmonella enterica* serovar Enteritidis (*S*. Enteritidis) is a pathogen that can colonize the preovulatory follicles of poultry, thereby causing both reduced egg production and an elevated risk of foodborne salmonellosis in humans. Although a few studies have revealed *S*. Enteritidis preferentially invades the granulosa cell layer within these follicles, it can readily persist and proliferate through mechanisms that are not well-understood. In this study, we characterized competing endogenous RNA (ceRNA) regulatory networks within duck granulosa cells following time-course of *S*. Enteritidis challenge. The 8108 long non-coding RNAs (lncRNAs), 1545 circular RNAs (circRNAs), 542 microRNAs (miRNAs), and 4137 mRNAs (fold change ≥2; *P* < 0.01) were differentially expressed during S. Enteritidis challenge. Also, eight mRNAs, eight lncRNAs and five circRNAs were selected and the consistent expression trend was found between qRT-PCR detection and RNA-seq. Moreover, the target genes of these differentially expressed ncRNAs (including lncRNAs, circRNAs and miRNAs) were predicted, and significantly enriched in the innate immune response and steroidogenesis pathways. Then, the colocalization and coexpression analyses were conducted to investigate relationships between ncRNAs and mRNAs. The 16 differentially expressed miRNAs targeting 60 differentially expressed mRNAs were identified in granulosa cells at 3 and 6 h post-infection (hpi) and enriched in the MAPK, GnRH, cytokine-cytokine receptor interaction, Toll-like receptor, endocytosis, and oxidative phosphorylation signaling pathways. Additionally, underlying lncRNA-miRNA-mRNA and circRNA-miRNA-mRNA ceRNA networks were then constructed to further understand their interaction during *S*. Enteritidis infection. Lnc_012227 and novel_circ_0004892 were identified as ceRNAs, which could compete with miR-let-7g-5p and thereby indirectly modulating *map3k8* expression to control *S*. Enteritidis infection. Together, our data thus identified promising candidate ncRNAs responsible for regulating *S*. Enteritidis infection in the preovulatory follicles of ducks, offering new insights regarding the ovarian transmission of this pathogen.

## Introduction

*Salmonella enterica* serovar Enteritidis (*S*. Enteritidis) is the primary *Salmonella* serovar isolated from poultry eggs, and it is frequently associated with egg-related foodborne disease outbreaks (1, 2). Duck consumption is second only to that of chickens in Asian nations (3, 4), with duck eggs accounting for an estimated 30% of all egg use in China and Southeast Asia (5). Epidemiological studies suggested *S*. Enteritidis infections to be commonplace in duck farms and slaughterhouses (6, 7), with infected ducks posing a substantial risk as sources of contaminated eggs, vertical *S*. Enteritidis transmission, and associated environmental damage.

*Salmonella* Enteritidis can colonize the preovulatory follicles in susceptible poultry, invading and multiplying within ovarian granulosa cells (8, 9). The ability of these cells to mount an effective immune response and maintaining normal physiological functionality is critical to the maintenance of ovulatory homeostasis and the avoidance of bacterial transovarial infections. *Salmonella* Enteritidis-infected chicken granulosa cells have revealed the pronounced upregulation of *tlr15, il-6, cxcli1, cxcli2*, and *k2033* at the mRNA level at 4 and 48 h post-infection (hpi) based on microarray analysis, whereas *rasd1* and *hb-egf* were significantly downregulated at these intervals. Underscoring the ability of *S*. Enteritidis infection to trigger an immune response in infected granulosa cells (10). *Salmonella* Enteritidis infection could additionally suppress steroidogenesis and impaired granulosa cell follicular growth, thereby reducing overall rates of ovulation and egg production (11). *Salmonella* Enteritidis could readily colonize duck granulosa cells (dGCs) and preovulatory follicles (12), yet the immune responses of dCGs from ducks following *S*. Enteritidis infection are poorly understood.

Non-coding RNAs (ncRNAs), including long ncRNAs (lncRNAs), microRNAs (miRNAs), and circular RNAs (circRNAs), have recently been shown to regulate diverse biological processes including immune responses to bacterial infection (13–16). miRNAs are able to suppress the translation of complementary target mRNAs (17), while circRNAs/lncRNAs can similarly interact with miRNAs to modulate their functionality. Indeed, the presence of shared miRNA response elements (MREs) among different RNA is thought to contribute to a competing endogenous RNA (ceRNA) cross-regulatory network within cells that can profoundly shape gene expression and host cell responses to diverse stimuli. Both the lncRNA-miRNA-mRNA and circRNA-miRNA-mRNA ceRNA networks are thought to orchestrate innate and adaptive immune responses to specific pathogens, with miRNAs playing a central role in this context as modulators of host-pathogen interactions (18). For example, a number of mammalian miRNAs (let-7, miR-15, miR-30, miR-128, miR-146, miR-155), lncRNAs (e.g., NeST, NEAT1v2), and circRNAs (mcircRasGEF1B) had been linked to *Salmonella* infection responses. During the *Salmonella* infection of murine macrophages, miR-21, miR-146a, and miR-155 had been strongly induce the activation of the transcription factor NF-κB and to inhibit B cell and T cell proliferation (19). Furthermore, let-7 family miRNAs could directly modulate the expression of key immunoregulatory cytokines including *il-6* and *il-10*, which hinted the downregulation of these miRNAs could increase the levels of these cytokines upon infection (20). LncRNA NeST could promote enhanced local IFN-γ production in mice, thereby aiding in the clearance of *Salmonella* (21). Similarly, mcircRasGEF1B regulates the LPS-TLR4-NF-κB by improving *icam-1* mRNA stability and reducing the severity of bacterial infection (13). Although these ncRNAs are well-studied in mammals, they are less well-understood in avian species. No prior studies have, to the best of our knowledge, explored the roles of lncRNA/circRNA-based ceRNA networks in the regulation of *S*. Enteritidis infection in ducks (*Anas platyrhynchos*).

In this study, we utilized a time-course *S*. Enteritidis infection model of dGCs to systematically identify patterns of differential circRNA, miRNA, mRNA, and lncRNA expression profile *via* high-throughput sequencing. We then constructed lncRNA-miRNA-mRNA and circRNA-miRNA-mRNA ceRNA networks to evaluate putative interactions between ncRNA and mRNA expression in this pathogenic context. Our data will provide a valuable foundation for the study of mechanisms regulating the control and ovarian transmission of *S*. Enteritidis infection in ducks.

## Materials and Methods

### Experimental Animals

Healthy Shaoxing ducks (*Anas platyrhynchos*, Chinese native breed, 26-weeks-old) free of *Salmonella* infection were obtained from the National Waterfowl Conservation Farm (Taizhou, Jiangsu, China). The Institutional Animal Care and Use Committee of the School of Animal Science and Technology, Yangzhou University approved all animal experiments in the present study (Permit Number: YZUDWSY, Government of Jiangsu Province, China).

### Duck Granulosa Cells Isolation and *S*. Enteritidis Infection

The isolation and culture of dGCs were performed as in previous reports (22, 23). Briefly, 10–15 pre-hierarchical follicles were isolated from Shaoxing ducks in the laying period under sterile conditions. Visible yolk and vitelline membrane were then finely minced, rinsed with PBS, and minced tissues were subsequently digested for 5 min with collagenase II (1 mg/mL; Gibco, NY, USA) at 37°C. A 200-μm sterile nylon mesh filter was then used to prepare cellular suspensions from these tissue digests, with filtered suspensions then being centrifuged two times (5 min, 67 × g).

Isolated pellets were then washed using M199 media (Hyclone, Utah, USA), resuspended in 3 mL of 50% Percoll, spun for 15 min at 421 ×g, and the cell layer was collected. The remaining dGCs were then suspended in M199 media containing 5% fetal calf serum, 2 mmol/L L-glutamine, 5 μg/mL transferrin, 10 μg/mL insulin, and 1.75 mM HEPES. Trypan blue exclusion was then used to evaluate cell viability, with only suspensions exhibiting > 90% viable cells being used for subsequent assays. These cells were cultured for 24 h in tissue culture flasks until fully adherent, with the GC-specific follicle-stimulating hormone receptor (FSHR) being used to assess cell purity *via* indirect immunofluorescence assay (IFA) with anti-FSHR (1:500, Proteintech, IL, USA, L594-22665).

*Salmonella* Enteritidis infections were conducted as previously reported (24, 25). Briefly, bacteria were cultured at 37°C to an OD_600_ of 2.0 (at mid logarithmic phase) in LB broth. Next, dGCs were plated in 96-well plates (1 × 10^5^/well) for 24 h, washed three times with PBS, and then treated with a 100 μL suspension of 10^6^ CFU *S*. Enteritidis in DMEM at a multiplicity of infection (MOI) of 10 for 1 h at 37°C, with control cells instead being treated with DMEM. Remaining non-invasive bacteria were then killed *via* the addition of DMEM containing 50 μg/mL gentamicin. After an additional 1 h incubation, assays were conducted in triplicate.

*Salmonella* Enteritidis infection was confirmed *via* an IFA approach as detailed previously (12). Briefly, media was removed and cells were washed three times with 0.01M PBS, fixed with 4% paraformaldehyde, permeabilized with 0.1% Triton X-100, and blocked for 1 h with 10% FBS in PBS. After a 2 h incubation with an anti-*Salmonella* antibody (1:2000, Abcam, MA, USA, ab69253), cells were washed thrice with PBS, stained with DAPI (0.2 mg/mL) for 15 min at 37°C, and imaged *via* fluorescence microscopy (Leica, Wetzlar, Germany).

### RNA Isolation, Small RNA Library Construction, and Sequencing

Trizol (Invitrogen, CA, USA) was used to isolate total RNA from 0, 3, and 6 hpi groups (*n* = 3, respectively), which was then treated with DNase I (Takara Biotechnology Co. Ltd., Dalian, China). A NanoDrop ND-2000 spectrophotometer (Thermo Scientific) was utilized to assess RNA quality based upon the A260/A280 ratio, with a value > 2.0 being consistent with high-quality RNA. A Qubit RNA Assay Kit and a Qubit 2.0 Fluorometer (Life Technologies, CA, USA) were used to quantify RNA levels in isolated samples, while an Agilent Bioanalyzer 2100 and an RNA 6000 Nano LabChip Kit (Agilent Technologies) were used to confirm RNA integrity (RIN), with a RIN number > 6 being sufficient for subsequent library construction.

A NEBNext Multiplex Small RNA Library Prep Set for Illumina (New England Biolabs, MA, USA) was used to prepare a small RNA library using 3 μg of total RNA per sample based on provided directions. Briefly, the NEB 3′ SR Adaptor was ligated to the 3′ ends of miRNAs, small interfering RNAs (siRNAs), and piwi-interfacing RNAs (piRNAs). An Illumina Hiseq 2,500 instrument (Illumina, CA, USA) was used to conduct 50 bp single-end reads of the resultant purified and enriched libraries.

### Strand-Specific Library Construction and Sequencing

In order to conduct lncRNA and circRNA sequencing, 3 and 5 μg of total RNA per sample were used, respectively. An Epicentre Ribo-zero rRNA Removal Kit (Epicentre, WI, USA) was used to remove rRNA from these samples, followed by ethanol precipitation. Next, 3 U of RNase R (Epicentre) per μg of RNA was applied to digest linear RNAs, and a NEBNext Ultra Directional RNA Library Prep Kit for Illumina (NEB) was used based on provided directions to construct sequencing libraries. Index-coded sample clustering was conducted with a cBot Cluster Generation System and the TruSeq PE Cluster Kit v3-cBot-HS (Illumina). Following cluster generation, an Illumina Hiseq 4,000 instrument was used to conduct 150 bp paired-end reads of prepared libraries.

### Sequencing Data Analyses

Custom Python and Perl scripts were utilized to clean raw FastQ files by removing reads containing adapter sequences, poly-N sequences, poly-A/T/C/G sequences, and low-quality reads with >50% of bases having a Q-score ≤ 10%. Raw data Q20 and Q30 scores, as well as GC content, were calculated. The resultant high-quality data were used for downstream analyses. The perl scripts used for the sequence quality control were from Novogene Gene Regulation Department.

The *Anas platyrhynchos* reference genome was downloaded from NCBI IASCAAS_PekingDuck_PBH1.5 (GCF_003850225.1), and a reference genome index was generated with Bowtie2 (26). Clean paired-end reads were then aligned to this reference genome with HISAT2 v2.0.4 (27), while Bowtie2 was used to map small RNA tags (26), and lncRNA and mRNA reads were assembled with StringTie (v1.3.1) (27), whereas find_circ was used to identify circRNAs (28). The small RNA tags were processed through miRBase20.0 to identify known *Gallus gallus* (closely related species) miRNAs. Potentially novel miRNAs were identified with miRDeep2 (29), which was used to generate predicted secondary structures and to predict miRNA precursor hairpin structures.

### Identification of Differentially Expressed RNAs

The transcripts per million (TPM) format was used to compare high-quality data (30), with the DESeq R package (1.10.1) being used to identify DE ncRNAs (31). For lncRNAs and mRNAs, Cuffdiff (v2.1.1) was utilized to calculate fragments per kilo-base millions of exon per million fragments mapped (FPKM) (32). FPKM values for a given gene were determined by adding together all transcript FPKM values for a given gene based on the following criteria: logFC > 2, FDR < 0.05. Those transcripts with an adjusted P < 0.05 were considered to be differentially expressed between groups using the Benjamini Hochberg method.

### Functional Enrichment Analyses

The GOseq R package was used to collect for gene length bias when conducting GO analyses (33), with GO terms yielding a corrected P < 0.05 being considered to be significantly enriched based Wallenius non-central hyper-geometric distribution. KEGG pathway enrichment for DE genes was assessed with the KOBAS software (34). Given their ability to regulate nearby protein-coding genes, GO and KEGG enrichment analyses of lncRNAs were additionally conducted using a 100 kb upstream/downstream colocalization threshold. Venn diagram analyses were used to identify pathways associated with up- or down-regulated coexpressed genes.

### ncRNA Regulatory Network Construction

Interactions between pairs of DE-lncRNAs and miRNAs were identified based upon sequence homology using the miRanda algorithm. The circRNA-miRNA-mRNA and circRNA-miRNA network interactions were predicted based upon the Circular RNA Interactome (https://circinteractome.nia.nih.gov/). LncRNAs/circRNA can competitively combine with the same miRNAs by MREs to suppress the inhibition of mRNA targeted by miRNAs and regulate the expression of the target genes.

Given that lncRNAs/circRNAs typically function as a molecular “sponge” that sequesters target miRNAs, lncRNAs/circRNAs often negative regulated miRNA expression. We selected circRNA-miRNA pairs regardless of the relative directionality of expression for these ncRNAs in analyzed samples. Significant DE-miRNAs and miRNA-mRNA pairs within these regulatory networks were identified. Potential DE-mRNA targets of DE-miRNAs were identified with the TargetScan database (https://www.targetscan.org), with those miRNA/mRNA pairs exhibiting inverse expression relationships being retained for network construction. These results were then used to construct final circRNA-miRNA-mRNA regulatory networks, which were visualized with Cytoscape 3.7.1 (https://cytoscape.org/).

### qPCR Detection for DE-ncRNAs and DE-mRNAs

The cDNA synthsis mixture for mRNA contained 1 μg of sample RNA, 4 μl of 5× FastKing-RT SuperMix and add RNA-free H_2_O up to 20 μl. The PCR reaction conditions included 42°C for 15 min and 95°C for 3 min. The cDNA synthsis for lncRNA and circRNA were used lnRcute lncRNA cDNA First-Strand Synthesis Kit (Tiangen, Beijing, China) and FastKing One Step for the First Chain Synthesis Kit (Tiangen, Beijing, China), respectively. Eight mRNAs, eight lncRNAs, and five circRNAs were selected for validation by qRT-PCR analysis. qRT-PCR was performed using the SYBR qPCR Master Mix (Vazyme). The amplification mixture (20 μl) contained 2 μl of cDNA, 0.4 μl of 10 μM each primer, 10 μl of SYBR qPCR Master Mix and 7.2 μl RNA-free H_2_O. The reaction conditions included 1 cycle at 95°C for 5 min, followed by 35 cycles of 95°C for 30 s, 60°C for 30 s, and 72°C for 30 s, and a final incubation at 72°C for 10 min. β*-actin* was used as reference gene. To confirm the miRNA transcriptome data, eight miRNAs were selected for qRT-PCR analysis. 3.75 μl of sample RNA was used to synthesize cDNA after adding a poly (A) tail to the 3′ end of the miRNAs using the Mir-X miRNA First-Strand Synthesis Kit (TAKARA). qRT-PCR was performed using the Mir-X miRNA qRT-PCR Kit (TB Green). The amplification mixture (25 μl) contained 2 μl of cDNA, 0.5 μl of ROX Dye, 0.5 μl of 10 μM each primer, 12.5 μl of 2 × TB Green Advantage Premix and 9 μl RNA-free H_2_O. The reaction conditions included 1 cycle at 95°C for 10 s, followed by 40 cycles of 95°C for 5 s, 60°C for 20 s, and a final dissociation at 95°C for 60 s, 55°C for 30 s and 95°C for 30 s. U6 snRNA was used as internal control to normalize miRNA expression studies (35). All Primers (Supplementary Table 1) were designed by Beijing Tiangen Co., Ltd. using Beacon Designer 7.9. The 2-^ΔΔ*Ct*^ method (36) was used to calculate relative expression.

### Luciferase Assay

The E1910 dual-luciferase reporter system (Promega, WI, USA) was utilized based on provided directions. Both miR-let-7g-5p mimic and negative control constructs were obtained from Shanghai GenePharma Co., Ltd. Wildtype luciferase reporter vectors (pMir-*map3k8*-3′UTR-WT, pMir-LNC_012227-WT, and pMir-novel_circ_0004892-WT) were constructed with the primers shown in Supplementary Table 2. Corresponding substitution mutant constructs (pMir-*map3k8*-3′UTR-MUT, pMir-LNC_012227-MUT, and pMir-novel_circ_0004892-MUT) were synthesized by Beijing Tsingke Co., Ltd. Renilla luciferase activity was used as a normalization control.

### Statistical Analysis and Data Available

All experiments were repeated in triplicate, and results were analyzed using independent sample *t*-tests (SPSS v 26, IBM, USA). The lncRNA-, circRNA-, and miRNA-seq data were deposited in the Short Read Archive (SRA) of the National Center for Biotechnology Information (NCBI) under the bio-project numbers PRJNA719952, PRJNA720231, and PRJNA720264.

## Results

### Duck Granulosa Cell Isolation and Infection With *Salmonella* Enteritidis Strain MY1

**Figure 1:**
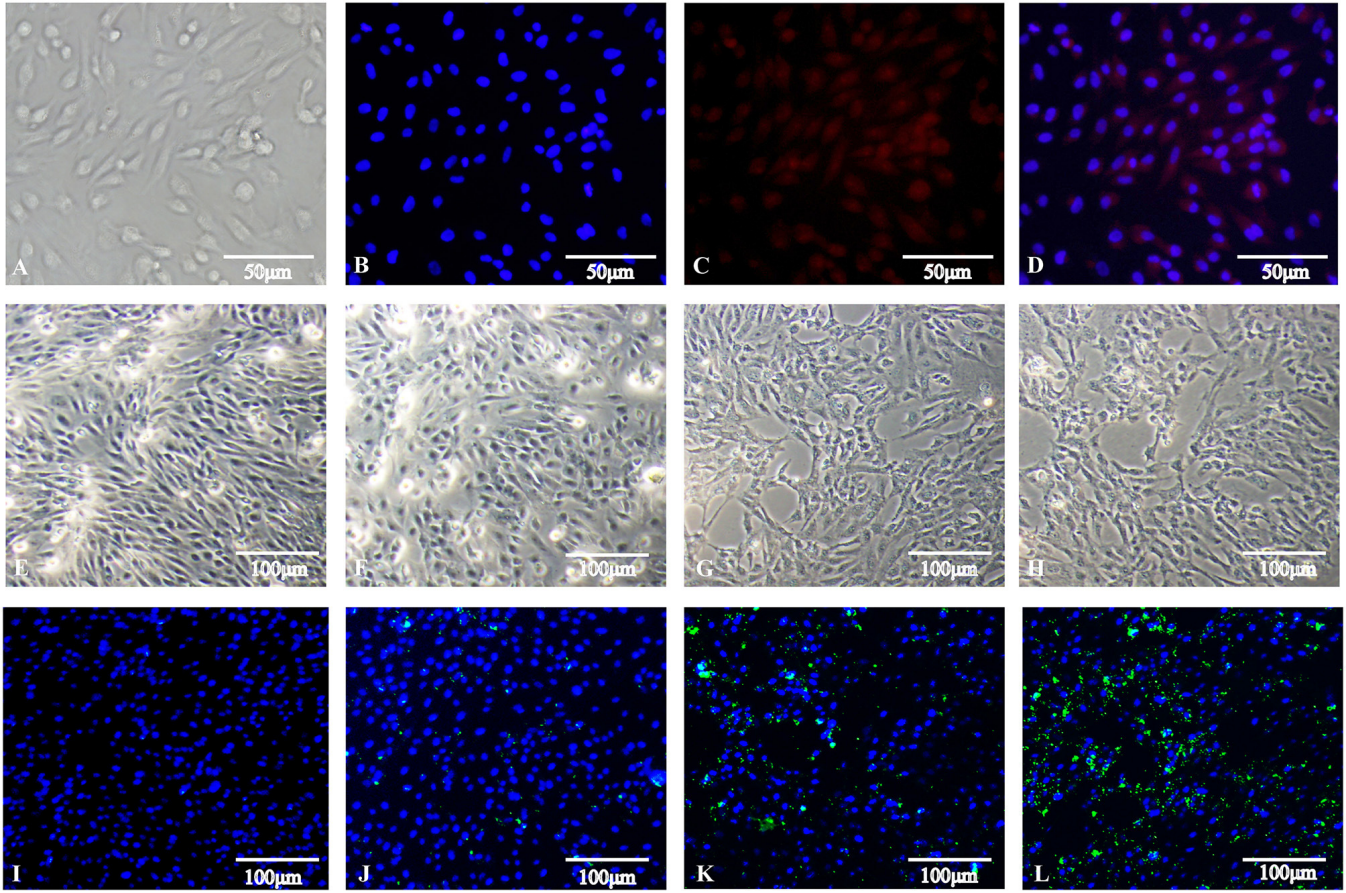
Isolation of duck granulosa cells and challenge with *Salmonella* Enteritidis MY1 strain. **(A–D)** Isolation and identification of duck granulosa cells. **(A)** Diagram of a duck granulosa cell (400×); **(B)** DAPI staining of cell nucleus; **(C)** Fluorescent image of FSHR; **(D)** Stacking chart of **(B,C)**. **(E–L)** dGCs morphology changes and the number of invasive SE at different times post-infection. Phase-contrast microscopy of dGCs infected at an MOI of 10 at 0 hpi **(E)**, 3 hpi **(F)**, 6 hpi **(G)**, and 9 hpi **(H)**, respectively. Indirect immunofluorescent staining and DAPI staining of dGCs infected at an MOI of 10 at 0 hpi **(I)**, 3 hpi **(J)**, 6 hpi **(K)**, and 9 hpi **(L)**, respectively.

After isolation, dGCs appeared round or ovoid in shape and adhered to tissue culture flasks in a monolayer with a pebble-like distribution within 24 h of culture. Cells were 100% confluent within 3–5 days, at which time granular material was evident in the cytoplasm. An IFA assay for the dGCs biomarker FSHR (red) was conducted to confirm the purity of these cells, with DAPI (blue) being used for nuclear counterstaining (Figures 1A–D). After infection and treatment with gentamicin as described in our section Materials and Methods, changes in cell morphology and the number of invasive *S*. Enteritidis were assessed at different time points. At 3 hpi, infected cells appeared swollen and irregular with some vacuolation, while at 6 hpi the cells were increasingly deformed and arranged in strip-shaped piles. By 9 hpi, many cells had begun to breakdown or dissolve, with the media appearing flocculated as a consequence of cellular detachment. An IFA staining assay revealed that dGCs invasion by *S*. Enteritidis increased significantly over time such that while few bacteria were detectable at 3 hpi, their numbers had grown exponentially by 6 hpi, and SE had invaded the majority of cells at 9 hpi (Figures 1E–L).

### Identification of *S*. Enteritidis-Related Patterns of Differential mRNA, miRNA, lncRNA, and circRNA Expression

**Figure 2:**
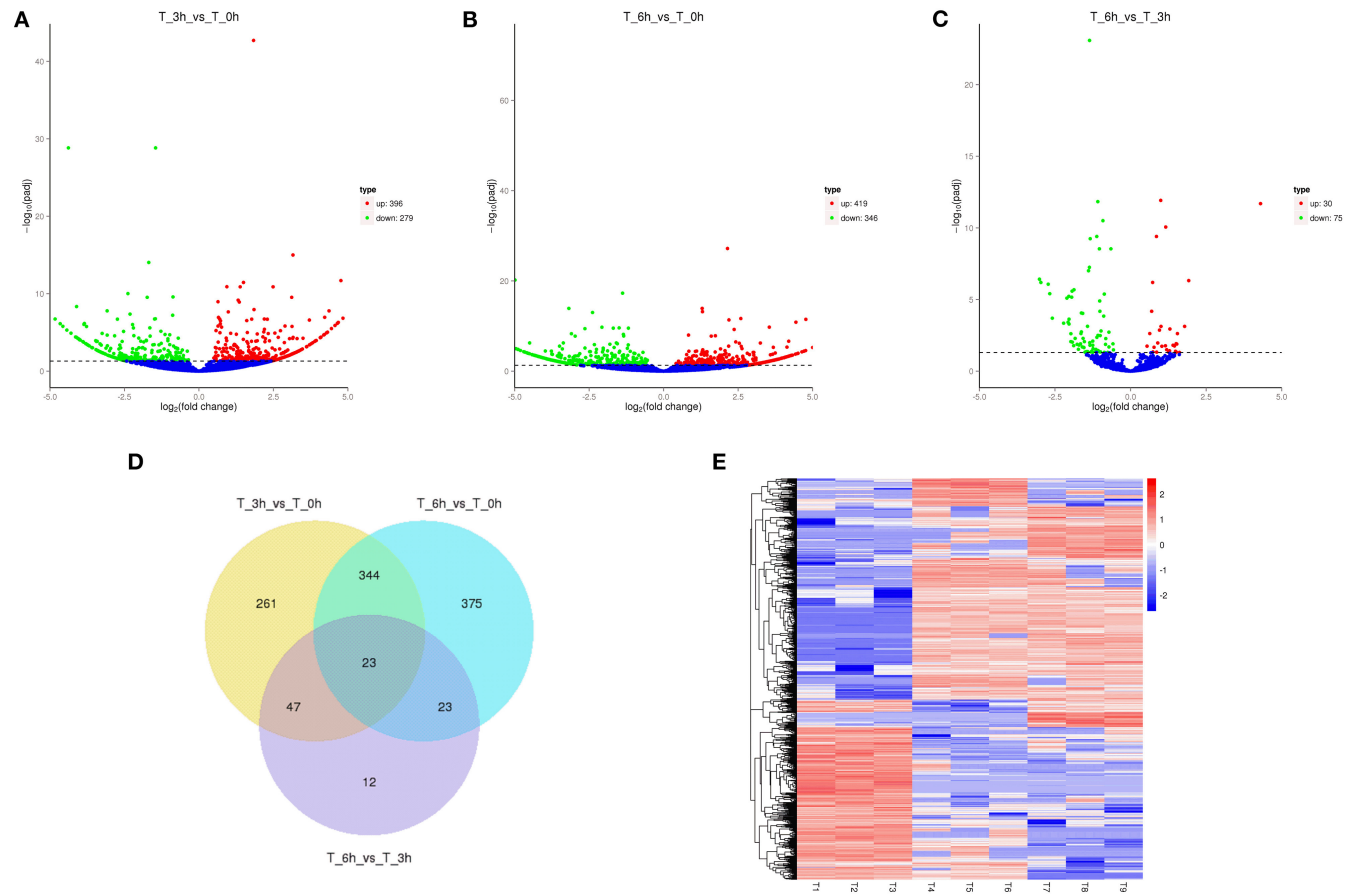
Changes in mRNA expression during SE infection in duck granulosa cells. **(A–C)** Volcano plots showing up- and down-regulated mRNAs of 3 vs. 0, 6 vs. 0, and 6 vs. 3 hpi group, respectively. **(D)** Venn diagram showing the number of overlapping differentially expressed mRNA between 0, 3, and 6 hpi. **(E)** Heat map of mRNAs showing hierarchical clustering of DE-mRNAs between 0, 3, and 6 hpi. Up- and down-regulated mRNAs are shown in red and blue, respectively.

**Figure 3:**
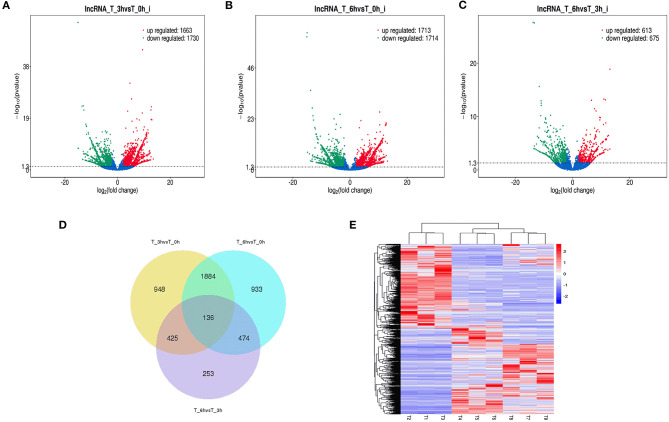
Changes in miRNA expression during SE infection in duck granulosa cells. **(A–C)** Volcano plots showing up- and down-regulated miRNAs of 3 vs. 0, 6 vs. 0, and 6 vs. 3 hpi group, respectively. **(D)** Venn diagram showing the number of overlapping differentially expressed miRNA between 0, 3, and 6 hpi. **(E)** Heat map of miRNAs showing hierarchical clustering of DE-mRNAs between 0, 3, and 6 hpi. Up- and down-regulated miRNAs are shown in red and blue, respectively.

**Figure 4:**
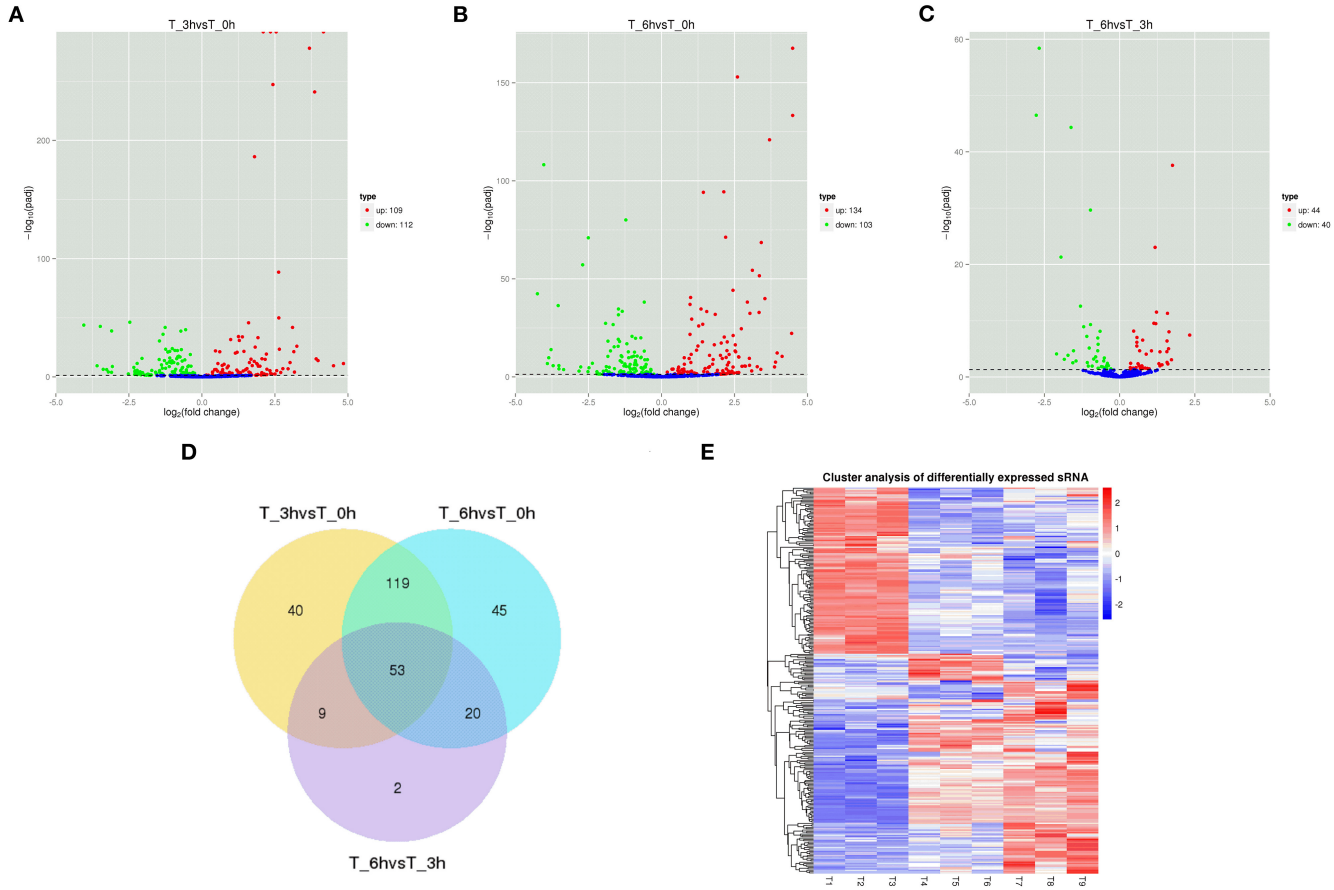
Changes in lncRNAs expression during SE infection in duck granulosa cells. **(A–C)** Volcano plots showing up- and down-regulated lncRNAs of 3 vs. 0, 6 vs. 0, and 6 vs. 3 hpi group, respectively. **(D)** Venn diagram showing the number of overlapping differentially expressed lncRNAs between 0, 3, and 6 hpi. **(E)** Heat map of lncRNAs showing hierarchical clustering of DE-mRNAs between 0, 3, and 6 hpi. Up- and down-regulated lncRNAs are shown in red and blue, respectively.

**Figure 5:**
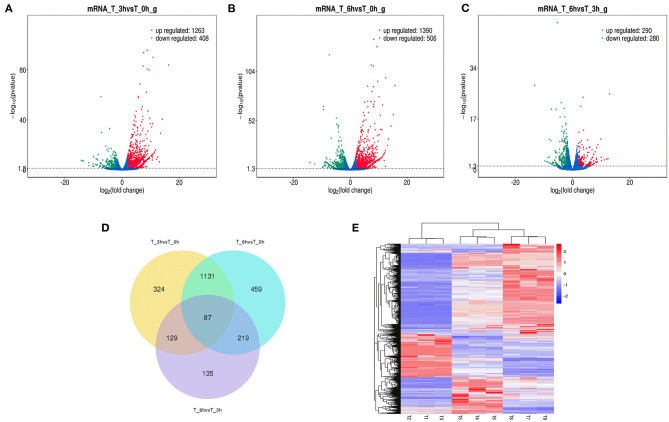
Changes in circRNAs expression during SE infection in duck granulosa cells. **(A–C)** Volcano plots showing up- and down-regulated circRNAs of 3 vs. 0, 6 vs. 0, and 6 vs. 3 hpi group, respectively. **(D)** Venn diagram showing the number of overlapping differentially expressed circRNAs between 0, 3, and 6 hpi. **(E)** Heat map of circRNAs showing hierarchical clustering of DE-mRNAs between 0, 3, and 6 hpi. Up- and down-regulated circRNAs are shown in red and blue, respectively.

High-level analyses of the mRNA-, miRNA-, lncRNA-, and circRNA-seq data generated at 0, 3, and 6 hpi were consistent with the high overall quality of these transcriptomic data (Supplementary Table 3). This led to the identification of 4,137 DE-mRNAs (2,943 up-regulated and 1,194 down-regulated), 542 DE-miRNAs (287 up-regulated and 255 down-regulated), 8,108 DE-lncRNAs (3,989 up-regulated and 4,119 down-regulated), and 1,545 DE-circRNAs (845 up-regulated and 700 down-regulated) in response to *S*. Enteritidis infection (Table 1), with full lists of these RNAs being compiled in Supplementary Tables 4–7. A series of Venn diagrams, cluster plots, and volcano plots were then used to assess patterns of differential mRNA and ncRNA expression across these three time points (Figures 2–5). Several of the identified DE-mRNAs were immune-related molecules such as pattern recognition receptors (*tlr2, tlr4, tlr5, tlr15, nlrc5*), T cell surface antigens (CD, CD36, CD80), and immune cell-derived cytokines (*il-6, il-8, il-10, il-12, ifn-*γ, *cxcr4*). Some of the identified miRNAs including let-7, miR-15, miR-146, miR-214, miR-29, and miR-128 have been shown to be important in the contest of *Salmonella* resistance, while miR-125b-5p, miR-34a-5p, miR-1416-5p, and miR-1662 have been shown to be differentially expressed in *Salmonella-*infected chickens. Many of the identified lncRNAs were related to immune processes, the top 10 most differentially expressed were LNC_012884, LNC_005648, LNC_005083, LNC_006273, LNC_014993, LNC_004863, LNC_004867, LNC_000117, LNC_000156, and LNC_000118 potentially controlling gene expression through the regulation of downstream target mRNAs. The top 10 most differentially expressed circRNAs identified as being potentially important in the context of immune responses to *S*. Enteritidis were novel_circ_0021339, novel_circ_0003348, novel_circ_0007574, novel_circ_0001064, novel_circ_0016980, novel_circ_0018123, novel_circ_0014419, novel_circ_0018388, novel_circ_0018338, and novel_circ_0000056.

**Table 1:** Summary of the number of differentially expressed ncRNAs and mRNAs.

**Group**	**Regulation**	**lncRNA**	**circRNA**	**miRNA**	**mRNA**
3 vs. 0 hpi	Up	1,663	396	109	1,263
	Down	1,730	279	112	408
6 vs. 0 hpi	Up	1,713	419	134	1,390
	Down	1,714	346	103	506
6 vs. 3 hpi	Up	613	30	44	290
	Down	675	75	40	280
Total		8,108	1,545	542	4,137

**Figure 6:**
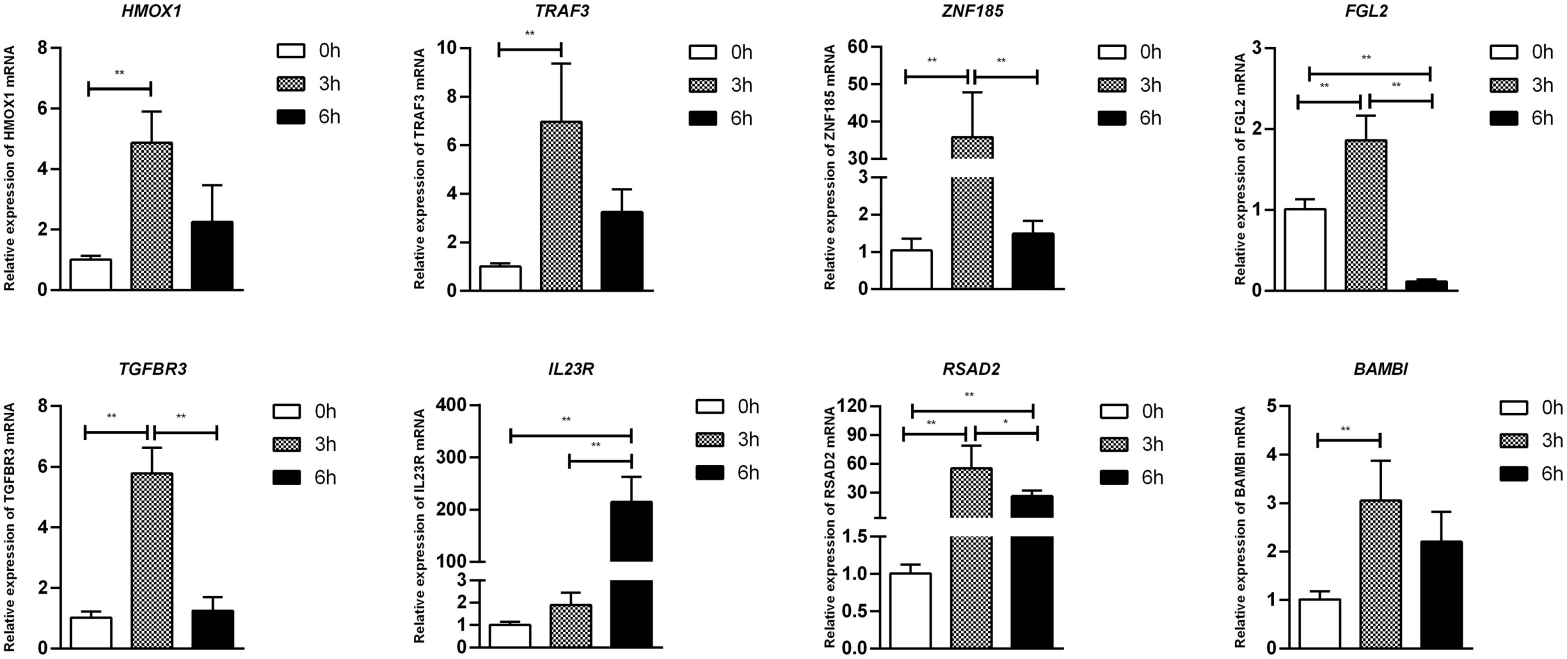
Validation of mRNA differential expression results during SE infection in duck granulosa cells at 0, 3, and 6 hpi. qRT-PCR validation of *HMOX1, TRAF3, ZNF185, FGL2, TGFBR3, IL23R, RSAD2*, and *BAMBI* mRNA expression levels in cell samples between 0, 3, and 6 hpi. The mRNA expression levels at 3 and 6 hpi were normalized to the value at 0 hpi. Error bars indicate the mean ± SD of triplicate experiments. β*-actin* was used as as reference gene. *P < 0.05; **P < 0.01.

**Figure 7:**
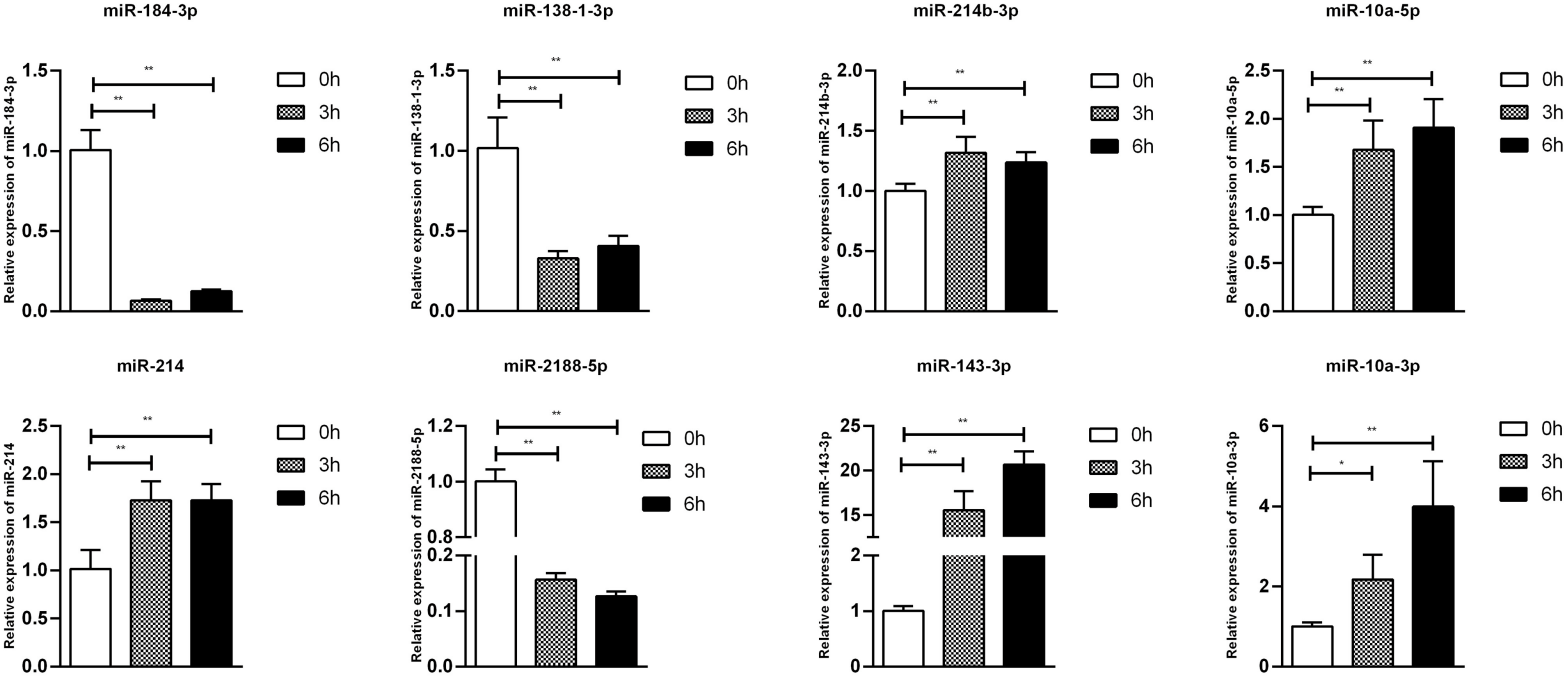
Validation of miRNA differential expression results during SE infection in duck granulosa cells at 0, 3, and 6 hpi. qRT-PCR validation of miR-2188-5p, miR-138-1-3p, miR-184-3p, miR-214b-3p, miR-10a-5p, miR-214, miR-10a-3p, and miR-143-3p miRNA expression levels in cell samples between 0, 3, and 6 hpi. The miRNA expression levels at 3 and 6 hpi were normalized to the value at 0 hpi. Error bars indicate the mean ± SD of triplicate experiments. U6 snRNA was used as internal control to normalize miRNA expression. *P < 0.05; **P < 0.01.

**Figure 8:**
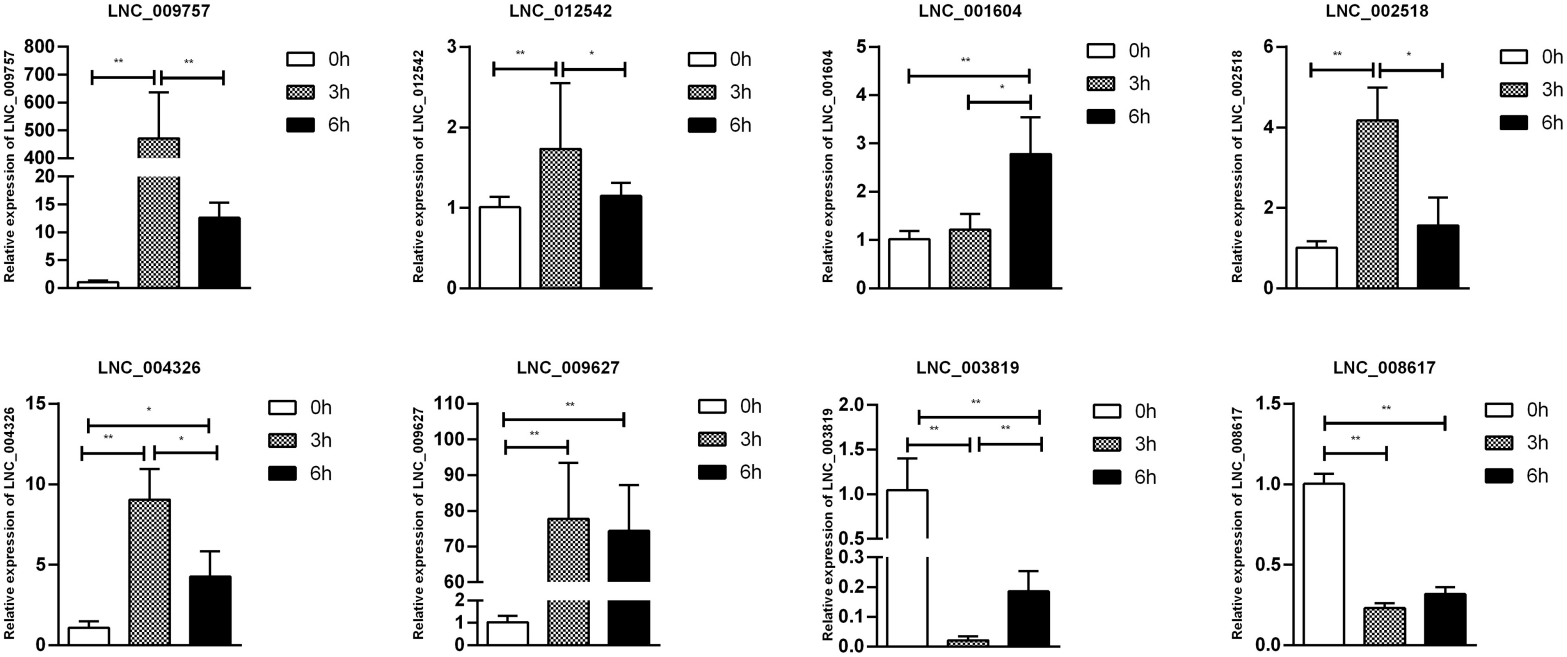
Validation of lncRNA differential expression results during SE infection in duck granulosa cells at 0, 3, and 6 hpi. qRT-PCR validation of LNC_002518, LNC_004326, LNC_009627, LNC_009757, LNC_012542, LNC_003819, and LNC_008617. lncRNA expression levels in cell samples between 0, 3, and 6 hpi. The lncRNA expression levels at 3 and 6 hpi were normalized to the value at 0 hpi. Error bars indicate the mean ± SD of triplicate experiments. β*-actin* was used as as reference gene. *P < 0.05; **P < 0.01.

**Figure 9:**
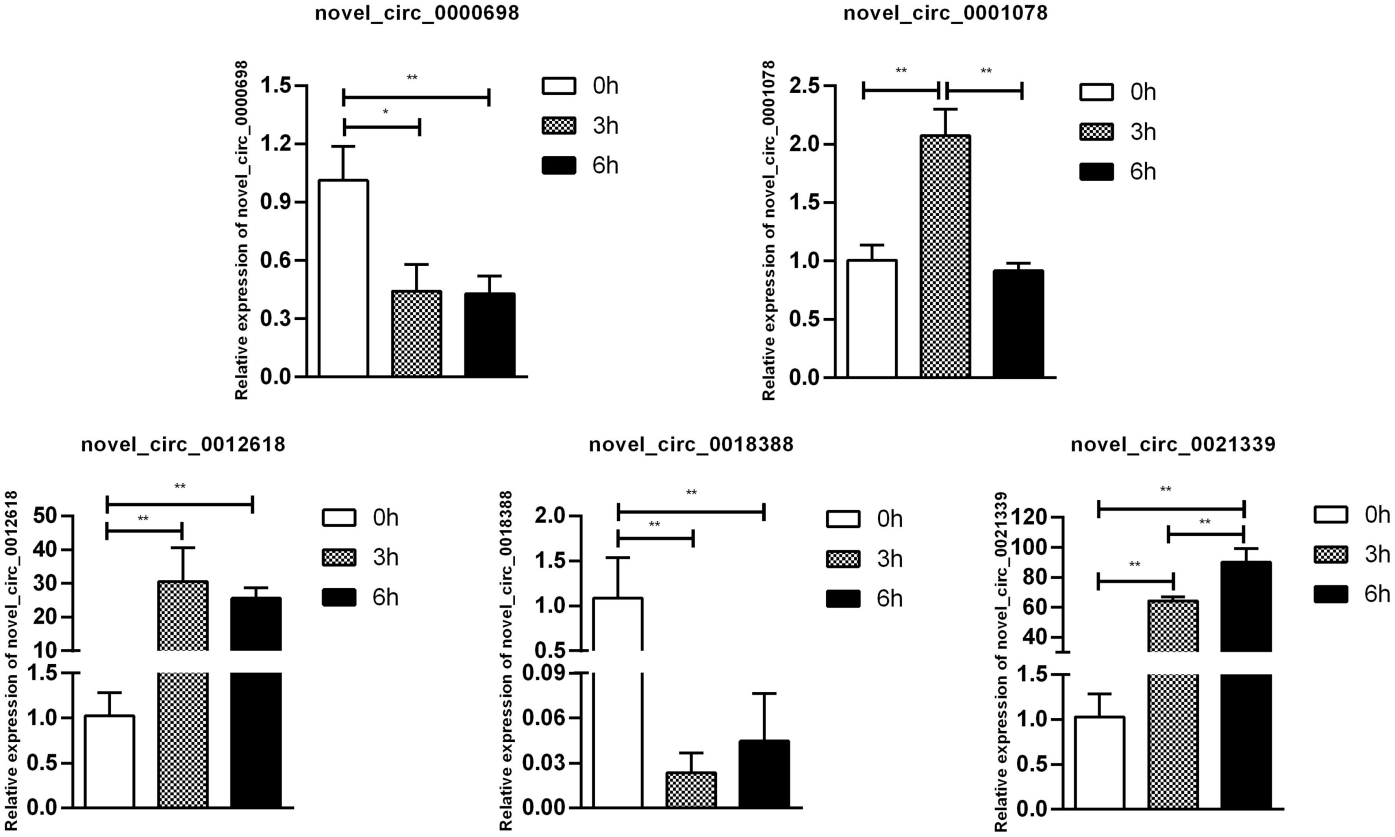
Validation of circRNA differential expression results during SE infection in duck granulosa cells at 0, 3, and 6 hpi. qRT-PCR validation of novel_circ_0018388, novel_circ_0021339, novel_circ_0012618, novel_circ_0001078, and novel_circ_0000698. circRNA expression levels in cell samples between 0, 3, and 6 hpi. The circRNA expression levels at 3 and 6 hpi were normalized to the value at 0 hpi. Error bars indicate the mean ± SD of triplicate experiments. β*-actin* was used as as reference gene. *P < 0.05; **P < 0.01.

To confirm furtherly the observe patterns of differential mRNA, miRNA, lncRNA, and circRNA expression in the context of *S*. Enteritidis infection, we next conducted a qPCR assay to validate the relative expression of 8 DE-mRNAs (*hmox1, traf3, znf185, fgl2, tgfbr3, il23r, rsad2*, and *bambi*), 8 DE-miRNA (miR-2188-5p, miR-138-1-3p, miR-184-3p, miR-214b-3p, miR-10a-5p, miR-214, miR-10a-3p, and miR-143-3p), 8 DE-lncRNAs (LNC_002518, LNC_004326, LNC_009627, LNC_009757, LNC_012542, LNC_003819, and LNC_008617), and 5 DE-circRNAs (novel_circ_0018388, novel_circ_0021339, novel_circ_0012618, novel_circ_0001078, and novel_circ_0000698) (Figures 6–9). The resultant qRT-PCR data were consistent with findings derived from our sequencing analyses.

### Pathway Enrichment Analyses

We next performed a GO enrichment analysis of DE-mRNAs identified in the present study (Supplementary Table 8), revealing them to be significantly enriched for terms including the immune response (GO: 0006955) and immune system process (GO: 0002376). The top 20 KEGG pathways related to these DE-mRNAs were additionally established (Supplementary Figure 1), and included the cytokine-cytokine receptor interaction, the Toll-like receptor signaling, *Salmonella* infection, and steroid hormone biosynthesis pathways that are linked to the regulation of immune responses, transcription, and signal transduction.

GO analyses of identified DE-miRNA target genes revealed these ncRNAs to be associated with processes including the cellular metabolic process (GO: 0044237), endosome (GO: 005768), and regulation of alpha-beta T cell proliferation (GO: 0046640) (Supplementary Table 9). The top 20 KEGG pathways associated with these DE-miRNAs were also identified (Supplementary Figure 2), and included physiological processes, signal transduction for steroid biosynthesis, the FoxO signaling pathway, and the mTOR signaling pathway.

This approach led to the identification of several immune response-related GO terms that were significantly enriched among the experimental groups (Supplementary Table 10), including the innate immune response (GO: 0045087), regulation of immune system process (GO: 0002682), immune system process (GO: 0002376), immune response (GO: 0006955), and positive regulation of innate immune response (GO: 0045089). The top 20 KEGG pathways related to these DE-lncRNAs at the three experimental time points were additionally identified based upon annotated functions of colocalized mRNAs (Supplementary Figure 3) and coexpressed mRNAs (Supplementary Figure 4), revealing significant enrichment for the Toll-like receptor, JAK-STAT, cytokine-cytokine receptor, and *Salmonella* infection signaling pathways.

Lastly, GO and KEGG analyses of the genes which gave rise to identified DE-circRNAs were conducted (Supplementary Table 11), revealing these genes to be enriched for immune response-related pathways including regulation of the immune system process (GO: 0002682), immune system development (GO: 0002520), immune response (GO: 0006955), the T cell receptor signaling pathway (GO: 0050852), and cytokine production (GO: 0001816). The top 20 KEGG pathways associated with DE-circRNA host genes when comparing samples at 0, 3, and 6 hpi were similarly associated with immune responses, and were enriched in the MAPK, JAK-STAT, and ECM-receptor interaction signaling pathways (Supplementary Figure 5).

### ceRNA Regulatory Network Construction

**Figure 10:**
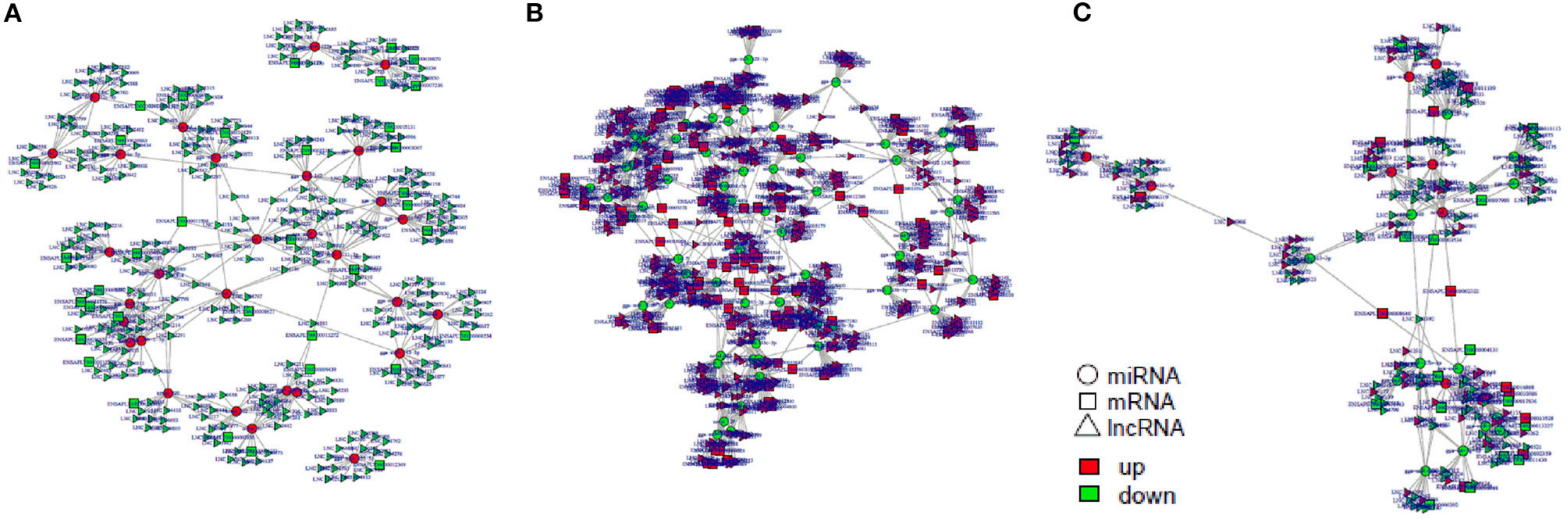
The regulatory networks of lncRNA-miRNA-mRNA during SE infection in duck granulosa cells. **(A)** lncRNA-miRNA-mRNA interaction network of 3 vs. 0 hpi group. **(B)** lncRNA-miRNA-mRNA interaction network of 6 vs. 0 hpi group. **(C)** lncRNA-miRNA-mRNA interaction network of 6 vs. 3 hpi group.

**Figure 11:**
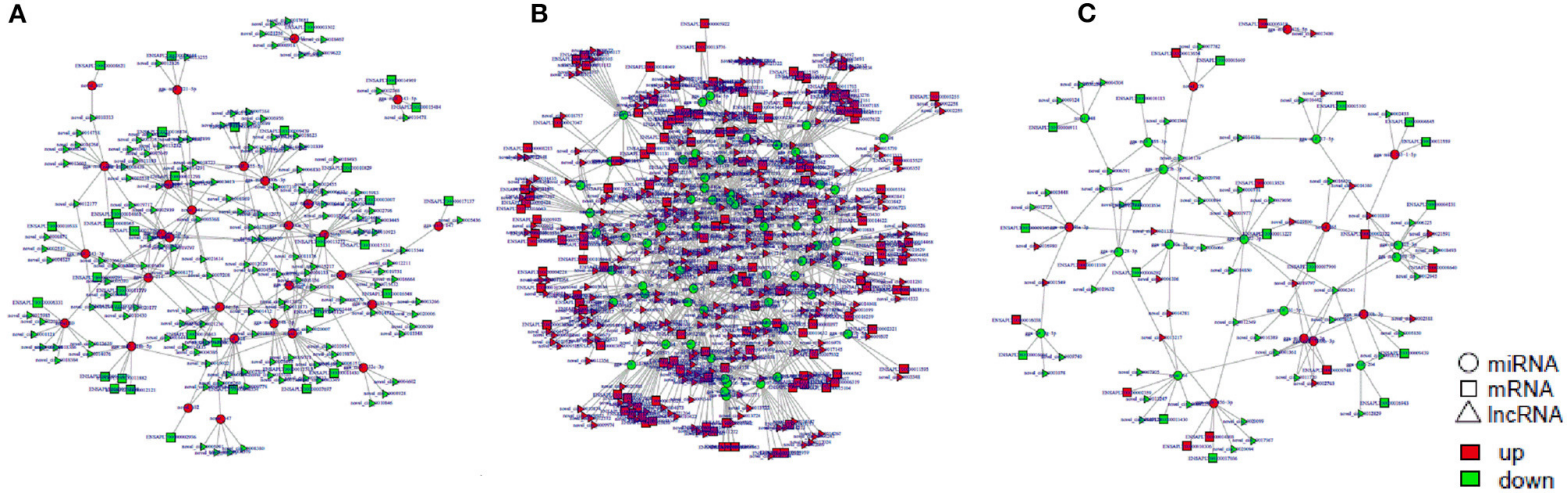
The regulatory networks of circRNA-miRNA-mRNA during SE infection in duck granulosa cells. **(A)** circRNA-miRNA-mRNA interaction network of 3 vs. 0 hpi group. **(B)** circRNA-miRNA-mRNA interaction network of 6 vs. 0 hpi group. **(C)** circRNA-miRNA-mRNA interaction network of 6 vs. 3 hpi group.

**Figure 12:**
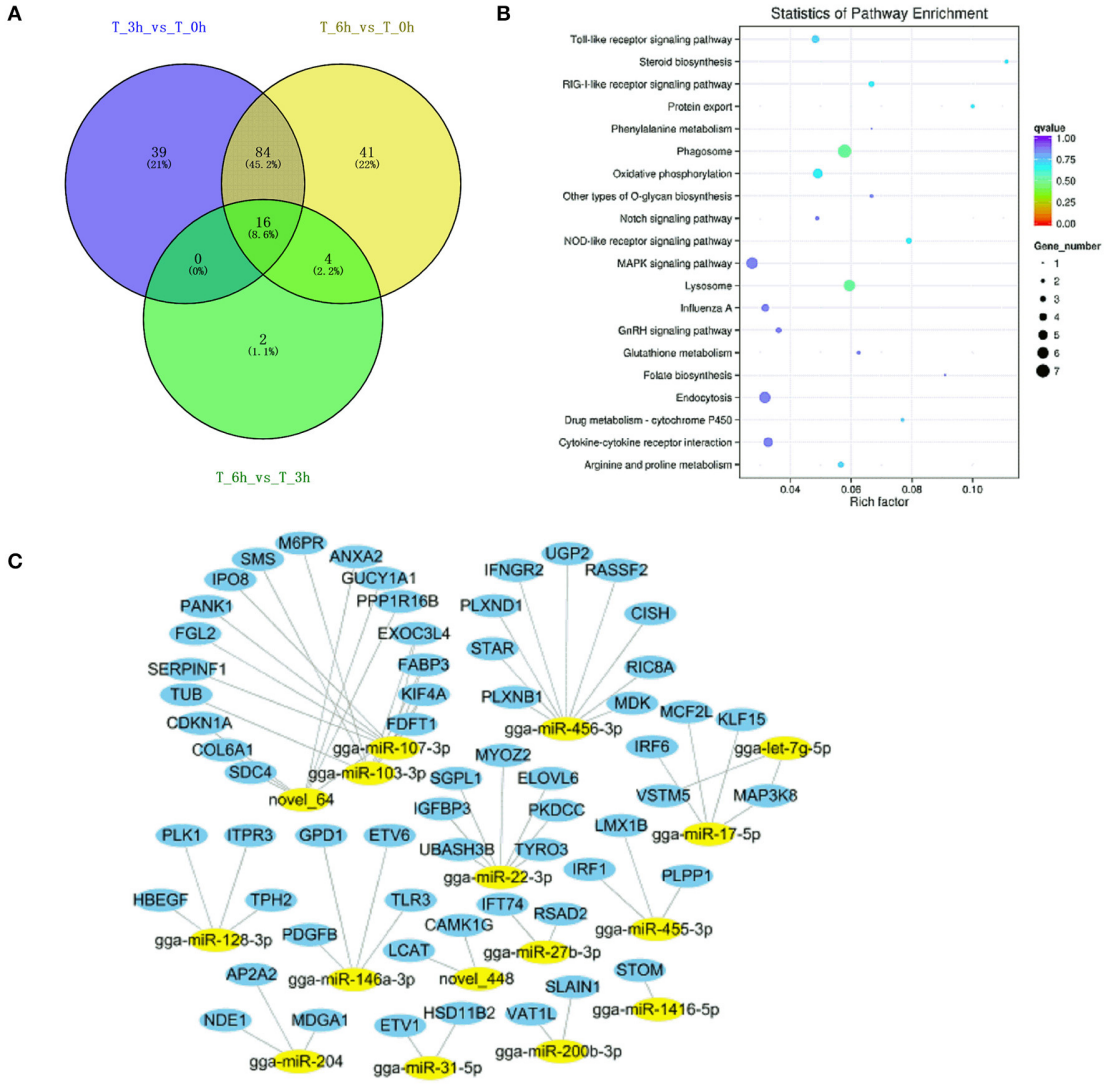
Analysis of targeted genes with significant differential expression miRNAs of lncRNA-miRNA-mRNA and circRNA-miRNA-mRNA regulatory networks. **(A)** DE-miRNAs were analyzed between 3 vs. 0, 6 vs. 0, and 6 vs. 3 h infection groups. **(B)** KEGG analysis of 16 DE-miRNAs targeted genes. **(C)** The regulatory network of 16 DE-miRNAs and targeted genes.

Next, we construct the relationship between mRNAs and ncRNAs within our dataset in order to better understand the factors governing immune responses to *S*. Enteritidis infection in dGCs. The ceRNA hypothesis posits that mRNAs and ncRNAs can compete for the complementary miRNAs, with the relative expression and localization of these RNA thereby regulating target gene expression. After analyzing DE-lncRNAs, DE-circRNAs, DE-miRNAs, and DE-mRNAs, we constructed a lncRNA-miRNA network containing 120,268, 141,522, and 12,193 lncRNA-miRNA pairs, including 1,798, 1,902, and 350 lncRNAs, and 221, 237, and 84 miRNAs by pairwise comparison 0, 3, and 6 time points, respectively (3 vs. 0, 6 vs. 0, and 6 vs. 3 hpi). We similarly identified 8,977 circRNA-miRNA pairs composed of 1,065 circRNAs and 288 miRNAs. Predicted miRNA target genes that were also represented within our DE-mRNA dataset were also identified, leading to the identification of 387, 495, and 33 potential miRNA-mRNA target pairs associated with our three comparison groups. Putative lncRNA-miRNA-mRNA and circRNA-miRNA-mRNA networks were then constructed based upon the above data (Figures 10, 11). The lncRNA-miRNA-mRNA networks contained 1,794, 1,899, and 331 lncRNAs, 139, 145, and 22 miRNAs, and 222, 289, and 27 mRNAs when comparing the 0, 3, and 6 hpi time points, respectively. The circRNA-miRNA-mRNA networks at these three respective time points included 629, 726, and 58 lncRNAs, 139, 145, and 22 miRNAs, and 222, 289, and 27 mRNAs. In these ncRNA-targeted mRNA of the ceRNA regulatory network, the *tlr3, il-6, il-8, il-22, inf-*γ, *cd74, smad3, map3k8, gata2*, and et al. were significantly upregulated, and *irf1, cd9, spsb1, ccl26*, and et al. were downregulated in *S*. Enteritidis infected dGCs (Supplementary Tables 12, 13). KEGG analysis was used further to study the function of ncRNA-targeted mRNA in the ceRNA regulatory network in *S*. Enteritidis infection. We found these mRNA to be primarily enriched for the Toll-like receptor signaling pathway and the cytokine-cytokine receptor interaction pathway at 3 and 6 hpi, respectively (Supplementary Figure 6). We additionally used the Venny 2.1 tool to analyze these ceRNA networks, leading to the identification of 16 core DE-miRNAs targeting 60 DE-mRNAs that were shared for the 3 vs. 0, 6 vs. 0, and 6 vs. 3 hpi comparison groups. KEGG analyses of these DE-mRNAs revealed them to be significantly enriched in eight pathways (*p* < 0.05), including the MAPK signaling, cytokine-cytokine receptor interaction, Toll-like receptor signaling, endocytosis, GnRH signaling, and oxidative phosphorylation pathways (Figure 12).

**Figure 13:**
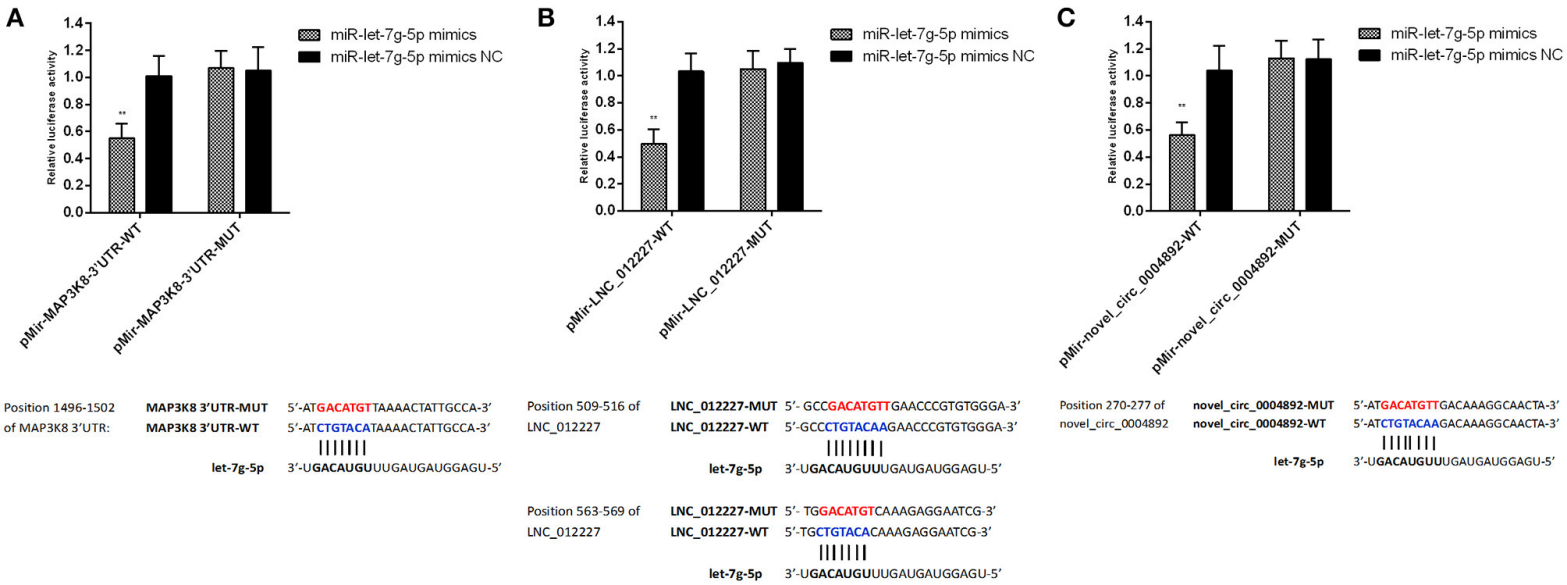
Verification of target binding. **(A)** miR-let-7g-5p putative binding sites on MAP3K8-UTR. Blue letters indicate wildtype sites and red letters indicate mutated sites in the pMir-report luciferase reporter vector. Luciferase assays took place in 293T cells co-transfected with pMir-report-MAP3K8-UTR-WT and miR-let-7g-5p mimics or pMir-MAP3K8-UTR-MUT and miR-let-7g-5p mimics. **(B)** Putative binding sites for miR-let-7g-5p on LNC_012227. Blue letters indicate wildtype sites and red letters indicate mutated sites in the pMir-report luciferase reporter vector. The luciferase assays took place in 293T cells co-transfected with pMir-LNC_012227-WT and miR-let-7g-5p mimics, or pMir-LNC_012227-MUT and miR-let-7g-5p mimics. **(C)** Putative binding sites for miR-let-7g-5p in novel_circ_0004892. Blue letters indicate wildtype sites and red letters indicate mutated sites in the pMir-report luciferase reporter vector. The luciferase assays took place in 293T cells co-transfected with pMir-novel_circ_0004892-WT and miR-let-7g-5p mimics, or pMir-novel_circ_0004892-MUT and miR-let-7g-5p mimics. Error bars indicate the mean ± SD of triplicate experiments. **P < 0.01.

Both LNC_012227 and novel_circ_0004892 were identified as ceRNAs for miR-let-7g-5p, which in turn targets *map3k8*. We employed a dual-luciferase reporter system to validate the predicted relationships between these RNAs, revealing that miR-let-7g-5p was able to suppress reporter luciferase activity by binding to complementary sequences in LNC_012227, novel_circ_0004892, and the *map3k8* 3′-untranslated region (Figure 13). These interactions between ncRNAs and the *map3k8* mRNA underscore the potential mechanisms whereby ncRNAs can shape dGCs physiological and immune responses in the context of *S*. Enteritidis infection.

## Discussion

*Salmonella* Enteritidis can be vertical transmission and contaminated eggs by colonization in avian reproductive tract and internalization in the forming egg. Some research suggests that *S*. Enteritidis attaches in distinct manners to developing and mature follicular granulosa cells, potentially contributing to ovary colonization and associated attachment in laying hens. Granulosa cells make up the final layer surrounding the yolk within preovulatory follicles, and function in concert with prehierarchical follicle functional differentiation to drive hierarchical development and egg yolk formation. Protective cellular immune responses play a key role in controlling *S*. Enteritidis infections while supporting sustained egg development.

Although a few investigation have revealed some circRNAs and miRNAs exerted function of immune responses in the spleens and cecum following *Salmonella* challenge in chickens (37–39). In this study, we found that 8108 lncRNAs and 1545 circRNAs were differentially expressed in ovary at 3 and 6 hpi during *S*. Enteritidis infection. Relative to mRNAs, lncRNAs are shorter, exhibit a lower GC content, harbor fewer exons, and exhibit incompletely understood functionality. By constructing lncRNA and mRNA co-expression networks, however, it is possible to predict potential lncRNA functions. When we constructed such networks and conducted KEGG analyses of the target genes contained therein, we found these genes to be primarily enriched for the Toll-like receptor signaling pathway and the cytokine-cytokine receptor interaction pathway at 3 hpi and 6 hpi, respectively. *Salmonella* Enteritidis infection was associated with the upregulation of *tlr3, il-6, il-8, il-22, inf-*γ, *cd74, smad3, map3k8*, and *gata2* and downregulation of *irf1, cd9, spsb1*, and *ccl26* in dGCs. Our data suggest a potential mechanism whereby lncRNAs may thus regulate immune responses at the post-transcriptional level, ultimately shaping the way cells respond to *S*. Enteritidis infection. Indeed, there is recent experimental evidence that lncRNAs can control immunity and host defense responses (16, 40). A prior time-course-based analysis of chicken granulosa cell responses to *S*. Enteritidis infection similarly revealed the upregulated expression of immune-related genes including *il-6, 8, inf-*γ, and *k60* (10). However, we observed significantly more DE-mRNAs associated with the immune system in our study of ducks relative to that prior report, with many of these DE-mRNAs being potential lncRNA targets. Supplementally, the sequencing data for infected cells at 9 hpi were excluded, as a large number of died cells appeared and their extracted RNA could not meet the requirements for RNA sequencing.

In the present study, we employed the colocalization and coexpression analyses to identify patterns of differential ncRNA expression and regulatory activity in the context of host immune responses to *S*. Enteritidis infection and ovarian transmission. Owing to the presence of shared MREs, lncRNAs, circRNAs, miRNAs, and mRNAs can all interact with one another to form complex ceRNA cross-regulatory networks that influence patterns of gene expression in a range of physiological and pathological contexts (41). The miRNAs (gga-miR-125b-5p, gga-miR-34a-5p, gga-miR-1416-5p, and gga-miR-1662), as mediators in the ceRNA network, had been also proved to result in the immune response to *S*. Enteritidis infection in hens (39). *Salmonella* infection is associated with reductions in nuclear RNA decay and with the upregulation of antibacterial ncRNAs (42). We specifically identified ncRNAs and mRNAs that were significantly differentially expressed at 3 or 6 h post-*S*. Enteritidis infection, revealing a greater degree of differential ncRNA expression at the later time point consistent with the gradual induction of cellular activity over the course of the progression of the *S*. Enteritidis infection.

In prior studies, lncRNAs and circRNAs have been shown to serve as ceRNAs to modulate miRNA-mediated post-transcription control of gene expression (37, 41). Several miRNAs are known to regulate innate immune responses induced by *S*. Enteritidis, such as gga-miR-155, gga-miR-1416-5p, gga-miR-1662, and gga-miR-34a-5p, which regulate the target genes *irf-4, tlr-21, bcl-10, tlr1la, notch2*, and *thbs1* (38, 39). When we performed KEGG pathway analyses of DE-miRNA target genes, we found them to be associated with metabolism and immune function. This is consistent with prior reports that miRNAs can fine-tune the metabolic processes engaged in the context of disease pathogenesis in a bidirectional manner (43, 44). The maintenance of a functional immune response is also highly dependent upon metabolic homeostasis. Our data further suggested that particular lncRNAs and circRNAs might interact with miRNAs and mRNAs to govern *S*. Enteritidis infection responses. The specific ceRNAs involved in regulating duck *S*. Enteritidis infection responses have not been published to date, and our high-throughput transcriptomic-based study thus provides novel insight regarding these dGCs ceRNA regulatory networks.

In order to validate the results of our ceRNA network analyses, we assessed LNC_012227 and novel_circ_0004892, and confirmed that both were able to function as molecular “sponges” for miR-let-7g-5p, which in turn targeted and suppressed the expression of *map3k8*. Other let-7 family miRNAs have been previously shown to be downregulated in the context of macrophage *Salmonella* infection, resulting in the upregulation of the cytokines *il-6* and *il-10* (20). IL-10 plays a deleterious role in chronic infections and limits microbial clearance in mice and humans (45). In addition, IL-10 can inhibit responses to acute infections or vaccines (46). Recently, Almanan et al. also found that IL-10 linked inflammation with immune suppression (47). In present study, IL10 family is up-regulated during *S*. Enteritidis challenge, which implied IL10 family might dampen responses to *S*. Enteritidis invasion resulting in persistence infection. We added some statement in the discussion section to explain the phenomenon of the IL10 family up-regulated. MAP3K8 is a serine/threonine kinase that can promote p38 MAPK and ERK1/2 phosphorylation, in addition to activating IKK and thereby promoting NF-κB nuclear localization and IL-2/TNF-α production in activated T cells. We observed high levels of *map3k8* expression in dGCs at 3 and 6 hpi, indicating that this kinase is likely an important regulator of cellular activity in the context of *S*. Enteritidis infection. MAP3K8 promotes inflammation by inducing cytokines, chemokines, and other inflammatory mediators mediated innate immunity against bacteria (48). The LncRNA LIPE-AS1 sponges miR-195-5p as a competitive endogenous RNA (ceRNA), which targets the 3′-untranslated region (3′-UTR) of MAP3K8 (49). Here, we also found Lnc_012227 and novel_circ_0004892 were identified as ceRNAs capable of sequestering miR-let-7g-5p and thereby indirectly modulating *map3k8* expression. The invasion of pathogenic bacteria in chicks is associated with MAPK signaling pathway activation (50), and p38 MAPK is also a key regulator of T cell survival and B cell maturation in inflamed tissues in humans (51). We identified LNC_012227 as being associated with *il-22* and *il-26*, which are important IL-10 family cytokines involved in STAT1, STAT3, MAPK1/3 (ERK1/2), JUN, and AKT activation and the induction of SOCS3 expression, ICAM1 surface expression, and the secretion of TNF-α, IL-8, and IL-10. ICAM1 is thought to regulate local innate immune responses, in addition to controlling cellular homeostasis during inflammation and promoting inflammatory responses (52, 53). The source gene of novel_circ_0004892 was identified as IL-1 receptor type 1-like (IL1R1), which is a receptor for the IL-1A, IL-1B, and IL-1RN cytokines. Following the binding of these cytokines, IL-1 associates with the IL1RAP co-receptor to generate a high-affinity IL-1R complex which activates the NF-κB, MAPK, and other signaling pathways (54). These pathways are dependent upon adapter proteins including TOLLIP, MYD88, and IRAK1 or IRAK2, interactive through TIR domains encoded by these different receptor subunits and adapter molecules. IL-1RN and other antagonistic molecules of similar affinity can bind to IL1RAP, preventing it from facilitating IL-1B-mediated co-stimulation of IFN-γ production from T-helper 1 (Th1) cells (55). Cells that express IL1-R1 are an important source of IL-22, which is in turn required for the coordination of effective responses against *Salmonella* owing to its ability to drive protective cytokine production and to prevent infectious colitis (56). Our results thus suggested that LNC_012227 and novel_circ_0004892 might be potent regulators of immune and metabolic responses in dGCs upon *S*. Enteritidis infection. However, more research will be required to establish the mechanisms whereby these ncRNAs regulate *map3k*8 expression and how these regulatory pathways shape *S*. Enteritidis pathogenesis and transmission.

## Conclusion

In conclusion, we identified specific circRNAs and lncRNAs likely to function as ceRNAs in regulating dGCs responses to *S*. Enteritidis infection. The 16 core miRNAs and 60 target mRNAs were regarded as important moderators including the LNC_012227/circ_0004892-miR-let-7g-5p-*map3k8* ceRNA axis, which controls *S*. Enteritidis-related immune responses in this pathological context. These results provide a novel foundation for future studies of the mechanisms whereby lncRNAs/circRNAs govern *S*. Enteritidis ovarian transmission in egg-laying ducks.

## Data Availability Statement

The datasets presented in this study can be found in online repositories. The names of the repository/repositories and accession number(s) can be found in the article/Supplementary Material.

## Ethics Statement

The animal study was reviewed and approved by The Institutional Animal Care and Use Committee of the School of Animal Science and Technology, Yangzhou University approved all animal experiments in the present study (Permit Number: YZUDWSY, Government of Jiangsu Province, China).

## Author Contributions

QX and GC conceived and designed the experiments. GZ assisted in experimental design. YZ and XD performed the experiments. LH, WV, and ZC analyzed the data. YZ wrote the paper. All authors contributed to the article and approved the submitted version.

## Conflict of Interest

The authors declare that the research was conducted in the absence of any commercial or financial relationships that could be construed as a potential conflict of interest.

## Supplementary Material

Supplementary Figure 1 Top 20 KEGG pathways associated with differentially expressed mRNAs between 0, 3, and 6 hpi. **(A)** Scatterplot showing KEGG pathway enrichment between 3 and 0 hpi. **(B)** Scatterplot showing KEGG pathway enrichment between 6 and 0 hpi. **(C)** Scatterplot showing KEGG pathway enrichment between 6 and 3 hpi.

Supplementary Figure 2 Top 20 KEGG pathways associated with differentially expressed miRNAs between 0, 3, and 6 hpi. **(A)** Scatterplot showing KEGG pathway enrichment between 3 and 0 hpi. **(B)** Scatterplot showing KEGG pathway enrichment between 6 and 0 hpi. **(C)** Scatterplot showing KEGG pathway enrichment between 6 and 3 hpi.

Supplementary Figure 3 Top 20 KEGG pathways based on mRNA colocalization with differentially expressed lncRNAs between 0, 3, and 6 hpi. **(A)** Scatterplot showing KEGG pathway enrichment between 3 and 0 hpi. **(B)** Scatterplot showing KEGG pathway enrichment between 6 and 0 hpi. **(C)** Scatterplot showing KEGG pathway enrichment between 6 and 3 hpi.

Supplementary Figure 4 Top 20 KEGG pathways based on mRNA coexpression with differentially expressed lncRNAs between 0, 3, and 6 hpi. **(A)** Scatterplot showing KEGG pathway enrichment between 3 and 0 hpi. **(B)** Scatterplot showing KEGG pathway enrichment between 6 and 0 hpi. **(C)** Scatterplot showing KEGG pathway enrichment between 6 and 3 hpi.

Supplementary Figure 5 Top 20 KEGG pathways associated with differentially expressed cirRNAs between 0, 3, and 6 hpi. **(A)** Scatterplot showing KEGG pathway enrichment between 3 and 0 hpi. **(B)** Scatterplot showing KEGG pathway enrichment between 6 and 0 hpi. **(C)** Scatterplot showing KEGG pathway enrichment between 6 and 3 hpi.

Supplementary Figure 6 Top 20 KEGG pathways associated with ncRNA-targeted mRNA in the ceRNA regulatory network in S. Enteritidis infection. **(A)** Scatterplot showing KEGG pathway enrichment of LncRNA-miRNA paired mRNA between 3 and 0 hpi. **(B)** Scatterplot showing KEGG pathway enrichment of LncRNA-miRNA paired mRNA between 6 and 0 hpi. **(C)** Scatterplot showing KEGG pathway enrichment of LncRNA-miRNA paired mRNA between 6 and 3 hpi. **(D)** Scatterplot showing KEGG pathway enrichment of cirRNA-miRNA paired mRNA between 3 and 0 hpi. **(E)** Scatterplot showing KEGG pathway enrichment of cirRNA-miRNA paired mRNA between 6 and 0 hpi. **(F)** Scatterplot showing KEGG pathway enrichment of cirRNA-miRNA paired mRNA between 6 and 3 hpi.

Supplementary Table 1 Primers used in this study.

Supplementary Table 2 Primers used for construction of the luciferase reporter vector.

Supplementary Table 3 Summary of RNA sequencing for each sample.

Supplementary Table 4 Analysis of differentially expressed mRNAs between 0, 3, and 6 hpi.

Supplementary Table 5 Analysis of differentially expressed miRNAs between 0, 3, and 6 hpi.

Supplementary Table 6 Analysis of differentially expressed lncRNAs between 0, 3, and 6 hpi.

Supplementary Table 7 Analysis of differentially expressed circRNAs between 0, 3, and 6 hpi.

Supplementary Table 8 Gene ontology classification of differentially expressed mRNAs between 0, 3, and 6 hpi.

Supplementary Table 9 Gene ontology classification of differentially expressed miRNAs between 0, 3, and 6 hpi.

Supplementary Table 10 Gene ontology classification of differentially expressed lncRNAs between 0, 3, and 6 hpi.

Supplementary Table 11 Gene ontology classification of differentially expressed circRNAs between 0, 3, and 6 hpi.

Supplementary Table 12 Analysis of lncRNA-miRNA-mRNA regulatory networks during SE infection in duck granulosa cells.

Supplementary Table 13 Analysis of circRNA-miRNA-mRNA regulatory networks during SE infection in duck granulosa cells.
