# Supplementary figures and images for: Identification of Differentially Expressed Non-coding RNA Networks With Potential Immunoregulatory Roles During Salmonella Enteritidis Infection in Ducks
Source: Front Vet Sci. 2021 Jun 16;8:692501. doi: 10.3389/fvets.2021.692501 (PMC8242174; doi:10.3389/fvets.2021.692501)

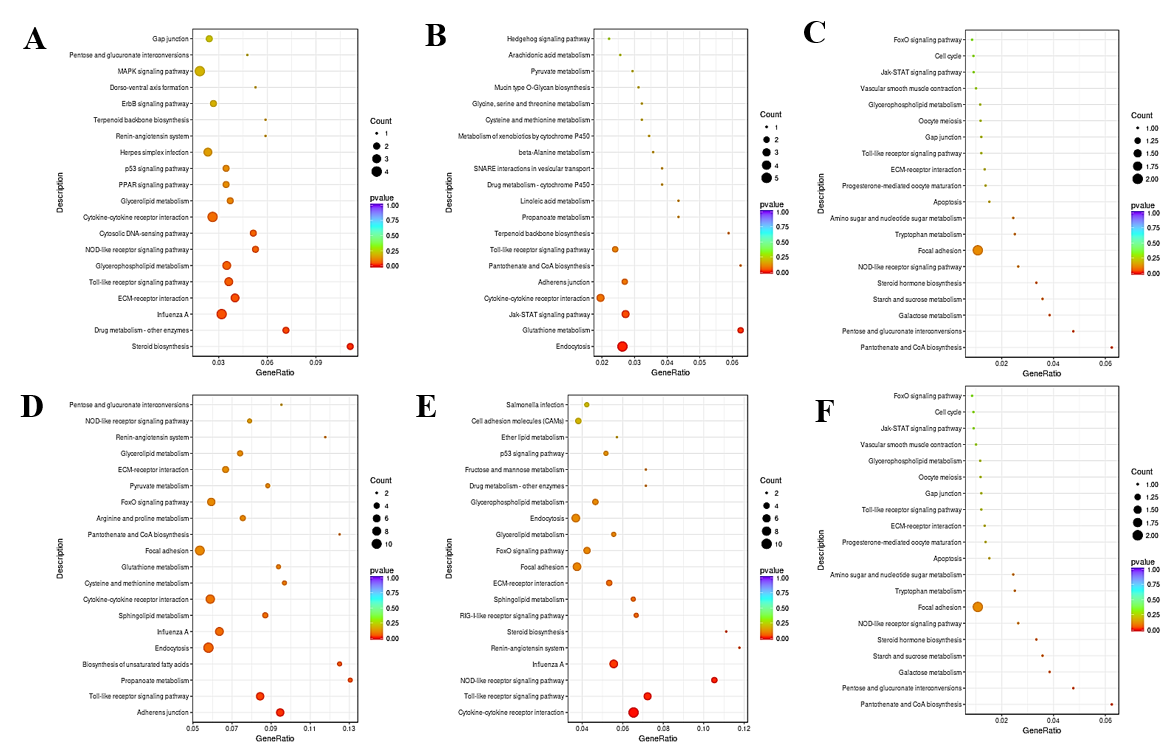

Supplement: Supplementary Figure 6 — Top 20 KEGG pathways associated with ncRNA-targeted mRNA in the ceRNA regulatory network in S. Enteritidis infection. (A) Scatterplot showing KEGG pathway enrichment of LncRNA-miRNA paired mRNA between 3 and 0 hpi. (B) Scatterplot showing KEGG pathway enrichment of LncRNA-miRNA paired mRNA between 6 and 0 hpi. (C) Scatterplot showing KEGG pathway enrichment of LncRNA-miRNA paired mRNA between 6 and 3 hpi. (D) Scatterplot showing KEGG pathway enrichment of cirRNA-miRNA paired mRNA between 3 and 0 hpi. (E) Scatterplot showing KEGG pathway enrichment of cirRNA-miRNA paired mRNA between 6 and 0 hpi. (F) Scatterplot showing KEGG pathway enrichment of cirRNA-miRNA paired mRNA between 6 and 3 hpi. [file Image_6.TIF]
